# Impact of Gestational Maternal SARS-CoV-2 Infection on Neonatal Inflammatory Biomarkers

**DOI:** 10.21203/rs.3.rs-8105673/v1

**Published:** 2025-12-22

**Authors:** Bushra Amreen, Floriana Milazzo, Frederieke Gigase, Darwin D’souza, Natalie Samper, Joseph Thomas Martin, Seunghee Kim-Schulze, Veerle Bergink, Jia Chen, Corina Lesseur, Anna-Sophie Rommel

**Affiliations:** Icahn School of Medicine of Mount Sinai; Icahn School of Medicine at Mount Sinai; Erasmus MC; Icahn School of Medicine at Mount Sinai; Icahn School of Medicine at Mount Sinai; Icahn School of Medicine at Mount Sinai; Icahn School of Medicine at Mount Sinai; Icahn School of Medicine at Mount Sinai; Icahn School of Medicine of Mount Sinai; Icahn School of Medicine of Mount Sinai; Icahn School of Medicine at Mount Sinai

**Keywords:** SARS-CoV-2, neonatal inflammation, prenatal infection, COVID-19, Olink^®^, Cytokines

## Abstract

**Background:**

Since the beginning of the pandemic, millions of pregnant women have been exposed to SARS-CoV-2, raising concerns about maternal and fetal sequelae. Yet, the impact of SARS-CoV-2 on the child’s immune response remains largely unexplored. Herein, we leverage 833 mother-infant dyads from a New York City-based pregnancy cohort, to explore prospective associations between maternal gestational SARS-CoV-2 infection and inflammatory biomarkers in newborns. Of the mothers, 100 were infected with SARS-CoV-2 during pregnancy, as confirmed through self-report, antibody and/or PCR test results. We obtained 92 inflammatory biomarker levels in neonatal dried blood spots (DBS) using the Olink^®^ Target 96 Inflammation panel. Empirical Bayes method was used to fit linear regression models to assess the effects of maternal infection during pregnancy on neonatal inflammatory markers at birth. We also conducted stratified analyses by timing of infection in early (<20 weeks) versus late (≥20 weeks) gestation.

**Results:**

Higher levels of 22 inflammatory biomarkers (*p*_*adj*_<0.05), including CD5, TNFSF14, CD8a, TGF-α, and CD244, were observed in neonates prenatally exposed to SARS-CoV-2 compared to unexposed neonates (*p*_*adj*_<*0.05*). Early-gestation infection was associated with increased levels of eight inflammatory biomarker, including TNSF14, TGF-α, EN-RAGE, and decreased IL-18 levels, while late-gestation infection was linked to elevations in 12 biomarkers, including CD5, CD6, PD-L1.

**Conclusion:**

Our results indicate that maternal SARS-CoV-2 infection during pregnancy impacts inflammatory biomarkers in newborns, with the timing of infection playing a critical role in shaping these immune profiles. Thus, this study underscores the need for further research and long-term follow-up to assess any potential future health consequences for the child.

## Background

Since the beginning of the COVID-19 pandemic, millions of pregnant individuals worldwide have been infected with the severe acute respiratory syndrome coronavirus-2 (SARS-CoV-2).^1^ Evidence from historical pandemics suggests that prenatal exposure to certain infectious agents, such as rubella and influenza, is associated with an elevated risk of adverse health outcomes in children born during those periods.^2^ Consistent with this pattern, gestational SARS-CoV-2 infection has been associated with adverse pregnancy and neonatal outcomes^3, 4^, although findings are heterogeneous.^5–7^ The overall risk of adverse maternal and neonatal outcomes appears greatest among individuals with symptomatic and severe infections^8–10^, with emerging evidence suggesting associations with neonatal complications, including respiratory distress.^11^ A recent meta-analysis further reported a substantially higher risk of respiratory distress syndrome (RDS) among neonates born to SARS-CoV-2 positive mothers^12^; and some studies suggested neurodevelopmental delays in children prenatally exposed to SARS-CoV-2. However, the evidence is inconsistent.^13–15^

Unlike other infectious agents, vertical transmission of SARS-CoV-2 occurs in only 1–3% of cases and viral placental infection is rarely reportedly.^16–19^ Nevertheless, even in the absence of neonatal infection, maternal SARS-CoV-2 infection seems to impact both the placenta and the fetus through placental immune activation and maternal vascular malperfusions.^20–24^ This is concerning because the development of the fetal immune system is orchestrated through carefully timed and sensitive stages starting from conception.^25, 26^ Any perturbation in this finely tuned developmental process may disrupt neonatal immune regulation and influence the child’s health outcomes down the line. Supporting this idea, animal studies have demonstrated that maternal inflammation alone, without direct viral transmission, can dysregulate cytokine levels in offspring.^27^ Additionally, studies also show that maternal immune activation or the trans-placental transfer of inflammatory mediators can shape the fetal immune system.^28, 29^

Research exploring the association between prenatal exposures to SARS-CoV-2 and neonatal immune response are sparse. Due to ethical and practical considerations^29^, cohorts that bank neonatal blood samples are rare and typically small. One approach to obtaining neonatal blood samples involves cord blood collection. As such, one study of 30 SARS-CoV-2 exposed and 15 unexposed mother-infant dyads measured multiple immune cell types and 13 cytokines in neonatal cord blood plasma, reporting elevated natural killer cells and regulatory T-cells in exposed neonates^30^. A more scalable and cost-effective method of obtaining and storing neonatal blood involves the use of dried blood spots (DBS). In one study, 42 cytokines/chemokines were measured in DBS from 460 neonates born to SARS-CoV-2 positive mothers and 85 neonates born to SARS-CoV-2 negative mothers, with IL-22 and GM-CSF showing significantly higher levels in exposed neonates before multiple testing correction.^31^ While these studies provide important early insights into the impact of prenatal SARS-CoV-2 exposure on the neonatal immune response, it remains challenging to form a comprehensive picture of the mechanisms at play, due to the variability in study design and findings. These discrepancies arise from the use of different biological matrices for measuring inflammatory markers such as DBS versus cord blood, as well as differences in maternal infection severity and definition of infection timing. Comparability is further limited by the minimal overlap in the specific inflammatory markers assessed in each study. Hence, additional studies are essential to strengthen the current body of evidence. In this study, we measured 92 inflammatory markers in neonatal DBS collected at birth in 100 children prenatally exposed to SARS-CoV-2 and 726 unexposed children. We explored associations between gestational SARS-CoV-2 exposure and neonatal inflammatory profiles, and further investigated whether these associations varied by timing of infection (early vs. late pregnancy).

## Methods

### Study population

**Figure 1:**
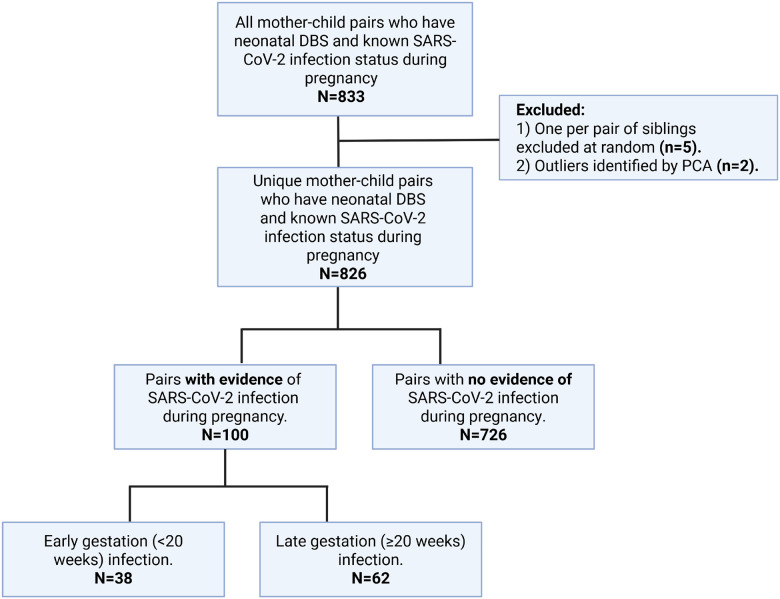
Flow chart of the final sub-cohort selection.

Between April 2020 and February 2022, the prospective cohort study Generation C recruited pregnant individuals (≥ 18 years) receiving obstetric care within the Mount Sinai Health System (MSHS). This cohort is described in detail elsewhere.^6^ For the present analysis, we examined a sub-cohort of 833 mother-infant dyads from the Generation C study. This sub-cohort comprised all mother-child pairs with known maternal SARS-CoV-2 infection status during pregnancy and consent for neonatal dried blood spot (DBS) retrieval. Five sibling pairs were included in this sub-cohort. For the analysis, if both siblings were unexposed to SARS-CoV-2 during gestation, one sibling per pair was excluded at random (Fig. 1). In cases where one of the siblings was exposed to SARS-CoV-2 during pregnancy, the unexposed sibling was excluded. All participants in this study provided informed consent. The study was approved by the Institutional Review Board (IRB-20–03352 and IRB-22–00566) at the Icahn School of Medicine at Mount Sinai, reviewed by the US Centers for Disease Control and Prevention (CDC), and conducted in compliance with relevant federal laws, CDC policies, and the Declaration of Helsinki.

### SARS-CoV-2 infection status and timing

In this study, maternal blood specimens were collected as part of routine clinical blood draws. A participant was considered to have evidence of SARS-CoV-2 if (1) there was a positive RT-PCR report or (2) there was a diagnosis by a medical health official reported in either the electronic medical record (EMR) or self-report questionnaire and (3) there was anti-S IgG antibody presence AND one of the following: a) anti-S IgG antibody before an individual’s first COVID-19 vaccination, b) anti-S IgG antibody before the COVID-19 vaccination rollout in NYC (Dec 14, 2020), or c) anti-spike IgG antibody presence and anti-N IgG antibody.^6^ For the first two scenarios, the date of diagnoses or report was considered the date of evidence of SARS-CoV-2 positivity while for the third, the date of sample collection was considered the date of positivity. If any of these dates were during pregnancy, a participant was considered to have evidence of SARS-CoV-2 exposure during pregnancy. To maximize power, we defined timing of infection as being infected early in gestation (< 20 weeks) and late in gestation (≥ 20 weeks), referred to below as early infection and late infection, respectively. Participants were considered unexposed if there was no evidence of SARS-CoV-2 positivity using any of the criteria above.

### Dried Blood Spot (DBS) collection and processing

Under the Newborn screening program (NBS), United States mandates collection of neonatal DBS at birth with the aim to screen for serious but treatable congenital diseases.^32^ Neonatal DBS were obtained from New York State Department of Health’s Newborn Screening Program (NYSDOH NBS). NYSDOH NBS collects five small blood spots by pricking the newborn’s heel, using a sterile lancet, within ~ 24 to 36 hours of delivery. The blood spots are collected on standard Whatman 903 contaminant free specimen cards. The spots are dried for at least three hours on a flat, clean, non-absorbent surface, away from direct heat and sunlight. Then residual DBS not used for clinical purposes are stored at room temperature for up to 27 years.^33^ We received six 3mm punches from each specimen card, one of which was eluted and incubated at room temperature for an hour, in 20ul of buffer, consisting of 1X PBS, 0.05% TWEEN 20, and 1X protease inhibitors. The eluted blood was used for further analysis.

### Olink^®^ Target 96 Inflammation panel

Neonatal inflammatory cytokines were quantified using the Olink^®^ Target 96 Inflammation Proteomics platform (Olink^®^ Bioscience, Uppsala, Sweden), with analyses conducted by the Human Immune Monitoring Center (HIMC) at Mount Sinai. Olink^®^ has become a widely adopted platform for large-scale proteomic analysis^34^ and has been leveraged in multiple DBS-based studies to date.^34–37^ This Olink^®^ Target 96 Inflammation panel employs a highly sensitive and specific proximity extension assay to quantitatively evaluate relative changes in the expression of 92 inflammation-related proteins.^38^ Briefly, pairs of oligonucleotide-conjugated antibodies, each recognizing a distinct target protein, were incubated with the samples. Upon concurrent binding to proximal epitopes, the antibody-bound oligonucleotides hybridized to form a double-stranded DNA template, which is subsequently amplified by PCR. The amplified DNA is then transferred to a microfluidic chip (Fluidigm BioMark HD instrument) and quantified using real-time quantitative PCR (qPCR). The raw data from the qPCR readout produces Cycle threshold (Ct) values, representing the number of amplification cycles required for the signal to reach a predetermined threshold. A lower Ct value indicates a higher initial concentration of the target protein. These Ct values are converted into Normalized Protein Expression (NPX) values, an arbitrary, relative unit on a log2 scale through a multistep process: 1) Normalization using Extension Controls: For each protein assay, the sample’s Ct value is subtracted from its corresponding Extension Control Ct value to yield ΔCt; 2) Inter-plate Normalization: The median ΔCt value for the Plate Control wells is subtracted from each sample’s ΔCt, resulting in a ΔΔCt value; 3) Final NPX Calculation: A pre-determined constant value (referred to as a correction factor) is subtracted from the ΔΔCt value to invert the scale so that a higher NPX value corresponds to a higher protein concentration, making the data more intuitive for biological interpretation. NPX values are on a log2 scale, meaning that a difference of one NPX represents a doubling of protein concentration. Because NPX values represent relative, sample-specific quantification, they are intended for group comparisons.^39^

### Covariates

Clinical and sociodemographic characteristics of interest were collected through EMR review and questionnaires, including maternal age (in years), maternal race/ethnicity (Asian, Black, Hispanic, White, Other), COVID-19 vaccination status (vaccinated, unvaccinated), pre-pregnancy BMI (kg/m^2^), cardiometabolic pregnancy complications (yes, no), preterm birth (yes, no), delivery mode (C-section, vaginal birth), child sex (male, female), age at DBS collection (in hours). COVID-19 vaccination status was defined as vaccinated if the first vaccine dose was administered before or during pregnancy. All mothers who were never vaccinated or those who were vaccinated after pregnancy were considered unvaccinated, as postnatal vaccination is unlikely to impact neonatal immune activation. Cardiometabolic pregnancy complications were defined as diagnoses of pre-eclampsia, gestational hypertension and/or gestational diabetes mellitus (GDM). COVID-19 symptom severity was determined using data from self-reported questionnaires and electronic medical records documenting concurrent symptom patterns, including fever or chills, cough, shortness of breath, fatigue, muscle or body aches, headache, loss of taste or smell, sore throat, congestion, nausea or vomiting, and diarrhea. Participants were categorized as having asymptomatic, mild, moderate, severe, or critical illness according to WHO COVID-19 clinical management guidelines^40^, based on symptom presentation, respiratory status, and clinical findings. Severity classification was applied only when symptoms were reported in the same trimester as the documented infection.

### Statistical analysis

To explore summary statistics of continuous and categorical variables, we used means, frequencies and ranges. We conducted bivariate analyses on sociodemographic and pregnancy outcome variables, using Chi-square test and Wilcoxon signed-rank, to compare how these variables differ between the SARS-CoV-2 exposed and unexposed groups. Correlations between NPX levels of the inflammatory markers were explored using Spearman’s rank-order correlation. To evaluate any possible effects of technical (e.g. assay batch) or biological (e.g. child sex) covariates on the inflammatory markers (NPX), we used principal component analysis. Any samples that were more than four standard deviations away from the mean on at least one principal component were excluded from the analysis. We excluded two motherchild pairs because their neonatal inflammatory cytokine measurements were identified as severe technical outliers **(Supplementary Fig. 1)**.

Differential analyses on inflammatory marker levels between SARS-CoV-2 exposed and unexposed groups were conducted using the Limma R package.^41^ This package uses an empirical Bayes method to fit linear regression models with moderated standard errors for each inflammatory marker as a continuous outcome and SARS-CoV-2 exposure status as dichotomous predictor variable. It also uses the Benjamini-Hochberg (BH) method to control the false discovery rate (FDR).

Additionally, to explore the impact of infection timing during pregnancy on neonatal inflammatory markers, we conducted a stratified sensitivity analysis by time of SARS-CoV-2 exposure during gestation. We stratified the overall SARS-CoV-2 exposed group into two groups, those exposed in early gestation (< 20 weeks) and those exposed in late gestation (≥ 20 weeks). We compared each group’s neonatal inflammatory marker levels independently to those of the unexposed group using Limma. Volcano plots were used to visualize log2-fold changes (Log2FC) in inflammatory marker levels for both overall and stratified analyses.

All final linear regression models were adjusted for maternal age, race/ethnicity, COVID-19 vaccination status, pre-pregnancy BMI, cardiometabolic pregnancy complications, preterm birth, delivery mode, child sex and age at DBS collection. All adjustment variables were categorical except maternal age, pre-pregnancy BMI and age at DBS collection. Statistical significance was set at ≤ 0.05 for nominal p-values and at 5% for false discovery rate (FDR). All analyses were conducted using R statistical computing software version 4.3.1.

## Results

### Cohort Characteristics:

After quality control and pre-processing, 826 mother-infant dyads were included in the study (Fig. 1). Table 1 describes the characteristics of the 826 mother-infant dyads, stratified by prenatal SARS-CoV-2 exposure. In this sample, 100 dyads had evidence of SARS-CoV-2 infection during pregnancy. Of these, 38 were exposed during early gestation (< 20 weeks) and 62 were exposed during late gestation (≥ 20 weeks). SARS-CoV-2 severity was classified as asymptomatic, mild, or moderate in 16%, 81%, and 3% of the exposed participants, respectively. In both the exposed and unexposed groups, mean maternal age was 33 years. Mean pre-pregnancy BMI was 27.5 kg/m^2^ (Standard Deviation (SD) = 6.6) and 26.7 kg/m^2^ (SD = 7.0) in the exposed and unexposed groups, respectively. Frequency of C-sections was similar between the exposed and unexposed group with vaginal birth being more prevalent in both. In the exposed group, 58.0% of the infants were female, compared to 50.0% of infants in the unexposed group. Mean gestational age at delivery in both groups was 39 weeks, with 12.0% (n = 12) and 10.1% (n = 73) preterm births in the exposed and unexposed groups, respectively. In the exposed group, 26.0% of the mothers had cardiometabolic pregnancy complications compared to 29.1% in the unexposed group. None of the aforementioned demographic and clinical variables were significantly different between the two study groups. However, we observed that exposed mothers were more likely to be Hispanic or Black compared to the unexposed group (*p = 0.04)*, on par with the larger cohort.^6^. Additionally, 33% of the exposed group was vaccinated before or during pregnancy, while 22.2% of the unexposed group was vaccinated before or during pregnancy (*p = 0.01)*.

### Differential Inflammatory marker expression analysis

**Figure 2:**
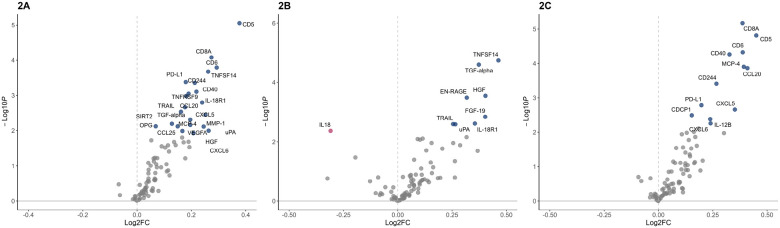
Volcano plots of differential inflammatory marker levels, in neonatal dried blood spots, using linear regression models adjusted for maternal age, race/ethnicity, vaccination status, pre-pregnancy BMI, preterm birth, delivery mode, infant sex, age at DBS collection and cardiometabolic pregnancy complications. **A.** Overall model of the exposed group (n=100) compared to the unexposed group (n=726). **B.** Model of the late infection group (n=62) compared to the unexposed group (n=726). **C.** Model of the early gestational exposure group (n=38) compared to the unexposed group (n=726). The x-axis is the log2-fold change (Log2FC) and y-axis is the –log10 P-value. The points highlighted in blue and maroon denote markers significant at FDR<0.05. Markers to the right of the vertical dashed line represent upregulation while markers to the left represent down regulation.

We conducted differential protein expression analyses by SARS-CoV-2 status using linear regression models comparing inflammatory marker levels of 100 exposed infants to 726 unexposed infants. In the exposed group, 22 neonatal inflammatory marker levels were increased (*p*_*adj*_<0.05) compared to the unexposed group. These include T-cell surface glycoproteins (CD5 and CD8A), T-cell differentiation antigen (CD6), Tumor necrosis factor receptor and ligand superfamily members (TNFSF14, TNFSF9 and CD40), TNF-related apoptosis-inducing ligand (TRAIL), C-C motif chemokines (CCL20, CCL25, MCP-4), C-X-C motif chemokines (CXCL6, CXCL5 and CXCL25), growth factors (TGF-α, HGF, VGFA) and several other cytokines, chemokines and growth factors (see Table 2 **&** Fig. 2A for details).

We used linear regression models to compare inflammatory marker levels of neonates exposed in early gestation (n = 38) and unexposed neonates (n = 726). Early infection analysis showed eight markers that were elevated (*p*_*adj*_<*0.05*) in the early infection group compared to the unexposed group, namely TGF-α, HGF, FGF-19, TNFSF14, TRAIL, RAGE-binding protein EN-RAGE, uPA and IL18R1. In contrast, interleukin-18 (IL-18) levels were lower (*p*_*adj*_<0.05) in the early infection group compared to the unexposed group (Table 3 & Fig. 2B).

Comparing inflammatory marker levels of neonates exposed to SARS-CoV-2 during late gestation (n = 62) and unexposed neonates (n = 726), twelve inflammatory markers showed differential expression (*p*_*adj*_<0.05). Namely, CD5, CD8A, CD6 and CD244, C-C and C-X-C motif chemokines (CXCL5 CXCL6, MCP-4, and CCL20), as well as CD40, PDL-1, CUB domain-containing protein 1 (CDCP1) and interleukin-12B (IL-12B) were elevated in the late infection group compared to the unexposed group (Table 4 & Fig. 2C).

**Figure 3:**
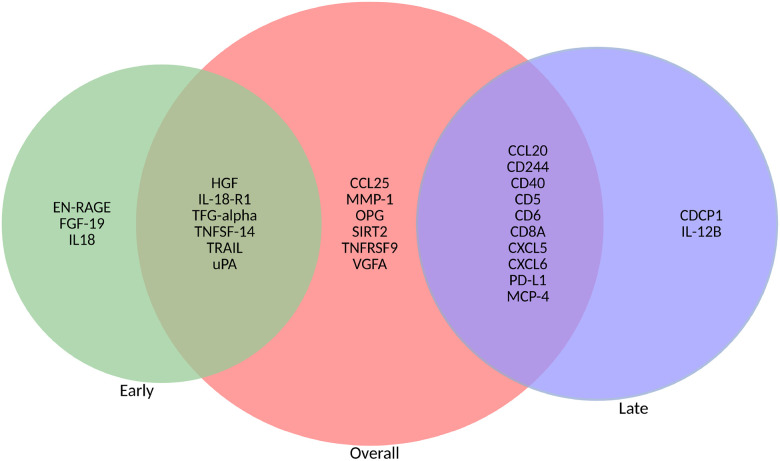
Venn diagram of overlapping inflammatory markers significant at FDR 5% early gestation, late gestation and overall analysis.

Figure 3 presents a Venn diagram illustrating the overlap in significant inflammation markers identified in the overall analysis and the stratified analyses. In general, the top significant inflammatory markers reaching 5% FDR in the overall analysis were also present in the early and late gestation analyses. Some markers in the overall analysis that do not overlap in Fig. 3 were within the 10% FDR significance threshold of the stratified analyses **(Supplementary Tables: 1, 2 & 3)**.

## Discussion

In the largest study of the association between prenatal exposure to SARS-CoV-2 and neonatal inflammatory profiles to date, we found that SARS-CoV-2 exposure during pregnancy influences inflammatory marker levels in the neonate. Several interesting markers and marker groups were differentially regulated in exposed compared to unexposed infants. Our data further revealed a striking divergence in immune responses based on timing of prenatal exposure to SARS-CoV-2. Whereas early gestational exposure was associated with post-inflammatory repair signatures and markers indicative of lung injury and recovery, late gestational exposure was associated with ongoing inflammatory signaling, as detailed below.

In neonates exposed to maternal SARS-CoV-2 infection during early gestation, we observed upregulation of pro-inflammatory proteins (EN-RAGE, TNFSF14, TRAIL, uPA), growth factors (HGF, FGF-19, TGF-α), and IL-18R, alongside reduced IL-18, suggesting enhanced IL-18/IL-18R1 binding. The discordant IL-18/IL-18R pattern points to NF-κB pathway activation,^42^ a key driver of inflammation in adult COVID-19^43^ and pediatric lung disease.^44^ TNFSF14 (LIGHT) has also been implicated in acute respiratory distress (ARDS) in hospitalized adult COVID-19 cases^45^ and virus-induced asthma exacerbation in children.^46^ In adults, growth factors, typically induced following lung injury, are associated with COVID-19 severity and tissue repair processes.^47–50^ Elevation of growth factors in neonates may indicate similar roles. Further, EN-RAGE, secreted by activated granulocytes, has also been linked to severe COVID-19 and impaired T-cell responses in adults.^51–54^ Elevated TRAIL in children, despite often being reduced in severe adult COVID-19^55^, may be reflective of distinct prenatal immune activation. Collectively, these patterns indicate that prenatal exposure to SARS-CoV-2, particularly during early gestation, may prime neonates toward inflammatory and tissue-repair responses, potentially reflecting a post-infection recovery phase rather than ongoing immune activation.

In contrast, neonates exposed to SARS-CoV-2 later in gestation exhibited a distinct immune profile characterized by upregulation of T-cell surface glycoproteins and Natural killer cell surface proteins (CD8A, CD5, CD44 and CD6), chemokines (CCL20, MCP-4, CXCL5 and CXCL6), interleukin IL-12B, and immune-checkpoint related proteins (CD40, CDCP1 and PD-L1). These markers are primarily linked to adaptive immunity, particularly CD8 + T-cells and TH1 responses, typically observed in asymptomatic or mild adult infections.^56, 57^ The chemokines elevated in this group mediate immune cell recruitment bridging innate and adaptive immune response. They also have been implicated in COVID-19 pathogenesis^57^ and the cytokine storm.^57–61^ Upregulation of PD-L1 and CD40, both associated with immune dysregulation and long COVID in children, further suggests altered immune signaling.^62, 63^ CDCP1, similarly elevated, has been linked to persistent post-infectious inflammation.^64^ It should be noted that increased T-cell surface markers maybe representative of higher of T-cell subpopulations in our exposed compared to unexposed neonates. Taken together, this constellation of markers indicates ongoing immune activation in late-exposed neonates, resembling patterns of active infection or post-infection hyperinflammation observed in adults and in multisystem inflammatory syndrome in children.

Expectedly, the observed patterns in the overall group align with the results described above for early and late gestation groups. For example, elevation of CCL25 in the overall group is in line with patterns in late gestation infection group, where we observe indication of ongoing immune activation. This is further supported by increased levels of immune markers like OPG, which has been associated with COVID-19 severity.^65^ Similarly, upregulation of VEGFA and MMP-1 is consistent with the upregulation of growth factors and lung injury-related proteinases in the early infection group^66, 67^ While, most markers increased in the overall analysis showed elevation in either the early or the late gestation exposure group, some markers, including CCL25, MMP-1 OPG, SIRT2, and VEGFA, were not clearly elevated in the smaller, time-stratified analyses. This discrepancy may be due to the limited statistical power in the time-stratified analyses. Supporting this possibility is that several of these markers, namely VEGFA, TNFRSF9, MMP-1, and SIRT2 in the early group, and OPG and CCL25 in the late group, met the 10% FDR threshold within their respective stratum.

These differences in neonatal immune profiles after early compared with late infection exposure may reflect the developmental stage of the fetal immune system at the time of exposure. The presence of adaptive immune markers (e.g., CD8A, IL-12B, PD-L1) in our late gestation-exposure group suggests prenatal immune priming. Given that vertical transmission of SARS-CoV-2 is rare, these immune signatures likely arise from maternal immune consequences in response to the SARS-CoV-2 infection or trans-placental transfer of inflammatory mediators rather than direct fetal infection.^28, 29^ Animal studies have shown that maternal inflammation can elevate offspring cytokine levels in the absence of viral transmission^27^, supporting this mechanism. Moreover, several upregulated proteins in our cohort, such as uPA and CD40, have been identified as biomarkers of long COVID in children, raising important questions about potential long-term immune and developmental consequences of prenatal exposure.

When compared to previously published studies, our results provide new insights into the neonatal inflammatory signatures associated with prenatal SAR-CoV-2 exposure. One previous study reported elevated IL-10 levels in cord blood plasma from neonates born to mothers with recent or ongoing infection compared to those who had recently recovered, with no significant differences in IL-12, GM-CSF, IFN and TNF levels.^30^ Beyond their smaller sample size and differences in exposure group classifications, the narrower and distinct cytokine profile reported in this study may reflect their exclusive focus on cord blood plasma, which captures soluble cytokines only. In contrast, our analysis of neonatal DBS, which contain both cellular and plasma components, enabled detection of cell-associated cytokines absent from plasma-only measurements. This distinction aligns with their cord blood mononuclear cell (CBMC) analyses, which identified alterations in particular T-cell subsets, patterns that resemble the elevated T-cell surface-related biomarkers we observed in the late gestation exposure group. Similar to the present analysis, another study investigated neonatal DBS and reported elevated cytokine levels (IL-22 and GM-CSF) prior to multiple comparison adjustment. Although both studies used DBS, methodological differences likely contributed to divergent findings. Specifically, Kim et al. quantified 42 cytokines/chemokines using the Luminex xMAP platform, which differs from Olink^®^ in sensitivity and target overlap.^30^ Additionally, differences in group balance between exposed and unexposed neonates (ours: 100 vs. 726; Kim *et al*.: 460 vs. 85) may have further contributed to the variability in study findings. Thus, our study extends existing evidence by integrating a larger sample, a broader proteomic panel, and cell-inclusive biospecimens to reveal timing-specific inflammatory signatures not captured in previous investigations.

Several limitations should be acknowledged. The number of participants differed between exposure groups, with a smaller sample size in the early gestation group (n = 38). Moreover, while the majority of dried blood spot (DBS) samples were collected within 24 hours of birth, a small subset was obtained later (> 24hr to 124hrs). To address these potential sources of variation, all models were adjusted for maternal vaccination status and the child’s age at DBS collection.

## Conclusions

Our study is among the first to examine a comprehensive panel of 92 neonatal inflammatory markers in a large cohort of children born to mothers infected with SARS-CoV-2 during pregnancy, and the first to assess how the timing of prenatal exposure influences inflammatory profiles at birth. Our results indicate that maternal SARS-CoV-2 infection during pregnancy impacts inflammatory biomarkers in the neonate, and that timing of infection plays a critical role in shaping these immune profiles. Thus, this study underscores the need for further research and long-term follow-up, to assess any potential future health consequences on the child.

## Supplementary Files

This is a list of supplementary files associated with this preprint. Click to download.

- SupplementaryMaterial.docx
- SUPPLEMENTALTABLES.xlsx

## Figures and Tables

**Table 1:** Population Characteristics mother child dyads (n = 826).

	Exposed to SARS-CoV-2 during pregnancy (N = 100)	Unexposed to SARS-CoV2 during pregnancy (N = 726)	*p*-value*
**Mother’s age at delivery**			
Mean (SD)	33.3 (4.69)	33.2 (5.07)	0.971
Median [Min, Max]	33.0 [21.0, 42.0]	34.0 [18.0, 50.0]	
**Child’s Gestational age at delivery**			
Mean (SD)	38.6 (1.77)	38.8 (1.89)	0.122
Median [Min, Max]	39.0 [32.9, 42.3]	39.1 [24.9, 42.0]	
**Preterm birth**			
Yes	12 (12.0%)	73 (10.1%)	0.671
No	88 (88.0%)	653 (89.9%)	
**Child sex**			
Female	58 (58.0%)	363 (50.0%)	0.163
Male	42 (42.0%)	363 (50.0%)	
**Delivery mode**			
C-Section	41 (41.0%)	285 (39.3%)	0.822
Vaginal	59 (59.0%)	441 (60.7%)	
**Cardio-metabolic pregnancy complications**			
Yes	26 (26.0%)	211 (29.1%)	0.605
No	74 (74.0%)	515 (70.9%)	
**Child Age at DBS collection (hours)**			
Mean (SD)	25.3 (12.1)	26.2 (10.5)	0.162
Median [Min, Max]	24.0 [1.00, 124]	25.0 [1.00, 110]	
**Pre-Pregnancy BMI (kg/m**^**2**^)			
Mean (SD)	27.7 (6.64)	26.7 (7.03)	0.114
**Mother’s age at delivery**			
Median [Min, Max]	26.1 [17.4, 45.4]	25.1 [14.2, 61.0]	
**Race/Ethnicity**			
Asian	6 (6.0%)	84 (11.6%)	0.044*
Black or African American	18 (18.0%)	94 (12.9%)	
Hispanic	35 (35.0%)	178 (24.5%)	
White	5 (5.0%)	37 (5.1%)	
Other	36 (36.0%)	333 (45.9%)	
**COVID-19 vaccination status**			
Unvaccinated	66 (66.0%)	565 (77.8%)	0.013*
Vaccinated	34 (34.0%)	161 (22.2%)	
**SARS-CoV-2 exposure timing**			
Early in gestation(< 20 weeks)	38 (38.0%)	0 (0%)	NA
Late in gestation (≥ 20 weeks)	62 (62.0%)	0 (0%)	
No SARS-CoV-2 infection	0 (0%)	726 (100%)	
**SARS-CoV-2 symptom severity**			
Asymptomatic	16 (16.0%)	0 (0%)	NA
Mild Illness	81 (81.0%)	0 (0%)	
Moderate Illness	3 (3.0%)	0 (0%)	
No SARS-CoV-2 infection	0 (0%)	726 (100%)	

* The p-values represented here are calculated through Chi-square test for categorical variables and Wilcoxon signed-rank test for continuous variables with nominal significance level set to p-value ≤ 0.05.

* SD = Standard Deviation; Min = Minimum; Max = Maximum;

**Table 2:** Differentially Regulated Inflammatory Markers in Overall Analysis, significant at 5% FDR.

Inflammatory Markers	Inflammatory Marker Description	Log2FC	*p*-value	*Adjusted p*-value
CD5	T-cell surface glycoprotein CD5	0.377	0.000	0.001
CD8A	T-cell surface glycoprotein CD8 alpha chain	0.274	0.000	0.004
CD6	T-cell differentiation antigen CD6	0.293	0.000	0.005
TNFSF14	Tumor necrosis factor ligand superfamily member 14	0.262	0.000	0.005
PD-L1	Programmed cell death ligand 1	0.179	0.000	0.007
CD244	Natural killer cell receptor 2B4	0.212	0.000	0.007
CD40	CD40L receptor	0.218	0.001	0.010
uPA	Urokinase-type plasminogen activator	0.190	0.001	0.010
TNFRSF9	Tumor necrosis factor receptor superfamily member 9	0.181	0.001	0.011
IL-18R1	Interleukin-18 receptor 1	0.240	0.002	0.015
TGF-α	Protransforming growth factor alpha	0.176	0.002	0.018
TRAIL	TNF-related apoptosis-inducing ligand	0.162	0.003	0.022
CCL20	C-C motif chemokine 20	0.252	0.004	0.025
CXCL6	C-X-C motif chemokine 6	0.196	0.005	0.032
OPG	Osteoprotegerin	0.128	0.006	0.037
HGF	Hepatocyte growth factor	0.196	0.007	0.037
SIRT2	NAD-dependent protein deacetylase sirtuin-2	0.069	0.008	0.037
CCL25	C-C motif chemokine 25	0.149	0.008	0.037
CXCL5	C-X-C motif chemokine 5	0.245	0.008	0.037
MMP-1	Matrix metalloproteinase-1	0.263	0.010	0.045
VEGFA	Vascular endothelial growth factor A	0.167	0.010	0.045
MCP-4	Monocyte chemotactic protein 4	0.207	0.012	0.050

**Table 3:** Differentially Regulated Inflammatory Markers in Early Infection model, significant at 5% FDR.

Inflammatory Markers	Inflammatory Marker Description	Log2FC	*p*-value	*Adjusted p*-value
TNFSF14	Tumor necrosis factor ligand superfamily member 14	0.463	0.000	0.001
TGF-α	Protransforming growth factor alpha	0.373	0.000	0.001
HGF	Hepatocyte growth factor	0.403	0.000	0.008
EN-RAGE	RAGE-binding protein (EN-RAGE)	0.318	0.000	0.008
FGF-19	Fibroblast growth factor 19	0.402	0.001	0.027
IL-18R1	Interleukin-18 receptor 1	0.355	0.002	0.030
TRAIL	TNF-related apoptosis-inducing ligand	0.255	0.003	0.030
uPA	Urokinase-type plasminogen activator	0.265	0.003	0.030
IL18	Interleukin-18	−0.309	0.004	0.044

**Table 4:** Differentially Regulated Inflammatory Markers in Late Infection model, significant at 5%FDR.

Inflammatory Markers	Inflammatory Marker Description	Log2FC	*p*-value	*Adjusted p*-value
CD8A	T-cell surface glycoprotein CD8 alpha chain	0.387	0.000	0.001
CD5	T-cell surface glycoprotein CD5	0.450	0.000	0.001
CD6	T-cell differentiation antigen CD6	0.388	0.000	0.001
CD40	CD40L receptor	0.327	0.000	0.001
MCP-4	Monocyte chemotactic protein 4	0.393	0.000	0.002
CCL20	C-C motif chemokine 20	0.409	0.000	0.002
CD244	Natural killer cell receptor 2B4	0.267	0.000	0.005
PD-L1	Programmed cell death ligand 1	0.198	0.002	0.019
CXCL5	C-X-C motif chemokine 5	0.352	0.002	0.023
CDCP1	CUB domain-containing protein 1	0.153	0.003	0.030
IL-12B	Interleukin-12 subunit beta	0.238	0.004	0.035
CXCL6	C-X-C motif chemokine 6	0.240	0.006	0.043

## Data Availability

Data will be made available upon request.

## References

[1] WHO. Coronavirus (COVID-19) Dashboard: Cases World Health Organization; 2025 [cited 2025]. Available from: https://data.who.int/dashboards/covid19/cases?n=o

[2] Lorea C. F., Pressman K. & Schuler-Faccini L. Infections during pregnancy: An ongoing threat. Semin Perinatol. 49 (4), 152075. 10.1016/j.semperi.2025.152075 (2025). Epub 20250406. 40189453

[3] Matsuo K., Green J. M., Herrman S. A., Mandelbaum R. S. & Ouzounian J. G. Severe Maternal Morbidity and Mortality of Pregnant Patients With COVID-19 Infection During the Early Pandemic Period in the US. JAMA Netw. Open. 6 (4), e237149. 10.1001/jamanetworkopen.2023.7149 (2023). Epub 20230403. PMC10082398 37027160

[4] Norman M. Association of Maternal SARS-CoV-2 Infection in Pregnancy With Neonatal Outcomes. JAMA 325 (20), 2076–2086. 10.1001/jama.2021.5775 (2021). 33914014 PMC8085767

[5] Du T., Zhang Y., Zha X. & Huang Q. Association of SARS-CoV-2 infection during late pregnancy with maternal and neonatal outcomes. BMC Pregnancy Childbirth. 24 (1), 632. 10.1186/s12884-024-06816-1 (2024). Epub 20241001. 39354438 PMC11446016

[6] Gigase F. A. J. SARS-CoV-2 infection, inflammation and birth outcomes in a prospective NYC pregnancy cohort. J. Reprod. Immunol. 163, 104243. 10.1016/j.jri.2024.104243 (2024). Epub 20240318. PMC11144074 38522364

[7] Sturrock S., Ali S., Gale C., Battersby C. & Le Doare K. Neonatal outcomes and indirect consequences following maternal SARS-CoV-2 infection in pregnancy: a systematic review. BMJ Open. 13 (3), e063052. 10.1136/bmjopen-2022-063052 (2023). Epub 20230315. PMC10030282 36921946

[8] Harel L. Does the presence of symptoms affect pregnancy outcomes in third trimester in women with SARS-CoV-2. J. Matern Fetal Neonatal Med. 35 (25), 7582–7589 (2022). 1956895. 34629031 10.1080/14767058.2021.1956895

[9] Metz T. D. National Institute of Child H, Human Development Maternal-Fetal Medicine Units N. Association of SARS-CoV-2 Infection With Serious Maternal Morbidity and Mortality From Obstetric Complications. JAMA 327 (8), 748–759. 10.1001/jama.2022.1190 (2022). 35129581 PMC8822445

[10] Metz T. D. Human Development Maternal-Fetal Medicine Units N. Disease Severity and Perinatal Outcomes of Pregnant Patients With Coronavirus Disease 2019 (COVID-19). Obstet. Gynecol. 137 (4), 571–580 (2021). doi: 10.1097/AOG.0000000000004339. 33560778 PMC7984765

[11] Man O. M. Respiratory distress in SARS-CoV-2 exposed uninfected neonates followed in the COVID Outcomes in Mother-Infant Pairs (COMP) Study. Nat. Commun. 15 (1), 399. 10.1038/s41467-023-44549-5 (2024). Epub 20240124. 38267411 PMC10808093

[12] Shabil M. Maternal COVID-19 infection and risk of respiratory distress syndrome among newborns: a systematic review and meta-analysis. BMC Infect. Dis. 24 (1), 1318. 10.1186/s12879-024-10161-1 (2024). Epub 20241119. 39563236 PMC11577808

[13] Fajardo-Martinez V. Neurodevelopmental delay in children exposed to maternal SARS-CoV-2 in-utero. Sci. Rep. 14 (1), 11851. 10.1038/s41598-024-61918-2 (2024). Epub 20240524. 38789553 PMC11126599

[14] Edlow A. G. Sex-Specific Neurodevelopmental Outcomes Among Offspring of Mothers With SARS-CoV-2 Infection During Pregnancy. JAMA Netw. Open. 6 (3), e234415. 10.1001/jamanetworkopen.2023.4415 (2023). Epub 20230301. PMC10037162 36951861

[15] Edlow A. G., Castro V. M., Shook L. L., Kaimal A. J. & Perlis R. H. Neurodevelopmental Outcomes at 1 Year in Infants of Mothers Who Tested Positive for SARS-CoV-2 During Pregnancy. JAMA Netw. Open. 5 (6), e2215787. 10.1001/jamanetworkopen.2022.15787 (2022). Epub 20220601. PMC9185175 35679048

[16] Zhang P. Maternal, neonatal and placental characteristics of SARS-CoV-2 positive mothers. J. Matern Fetal Neonatal Med. 35 (25), 5783–5791 (2022). 33645395 10.1080/14767058.2021.1892637

[17] Deniz M. & Tezer H. Vertical transmission of SARS CoV-2: a systematic review. J. Matern Fetal Neonatal Med. 35 (14), 2655–2662 (2022). 1793322. 32693656 10.1080/14767058.2020.1793322

[18] Cribiu F. M. Severe SARS-CoV-2 placenta infection can impact neonatal outcome in the absence of vertical transmission. J. Clin. Invest. 131 (6). 10.1172/JCI145427 (2021). PMC7954587 33497369

[19] Jeganathan K. & Paul A. B. Vertical transmission of SARS-CoV-2: A systematic review. Obstet Med. ;15(2):91 – 8. doi: 10.1177/1753495X211038157. (2022). 35795545 PMC9247633

[20] Keuls R. A. Single-nucleus transcriptional profiling of the placenta reveals the syncytiotrophoblast stress response to COVID-19. Am. J. Obstet. Gynecol. 232 (4S), S160–S75e7. 10.1016/j.ajog.2025.01.028 (2025). 40253079 PMC13281363

[21] Heeralall C. The effect of COVID-19 on placental functioning in South African pregnancies: investigation of kisspeptin expression and vascular and inflammatory alterations. Histochem. Cell. Biol. 163 (1), 49. 10.1007/s00418-025-02381-6 (2025). Epub 20250505. 40323370 PMC12053201

[22] Heeralall C., Ibrahim U. H., Lazarus L., Gathiram P. & Mackraj I. The effects of COVID-19 on placental morphology. Placenta 138, 88–96. 10.1016/j.placenta.2023.05.009 (2023). Epub 20230518. 37235921 PMC10191727

[23] Shanes E. D. Placental Pathology After SARS-CoV-2 Infection in the Pre-Variant of Concern, Alpha / Gamma, Delta, or Omicron Eras. Int. J. Surg. Pathol. 31 (4), 387–397 (2023). Epub 20220529. doi: 10.1177/10668969221102534. 35645148 PMC9152633

[24] Gabby L. C. Chronic villitis as a distinctive feature of placental injury in maternal SARS-CoV-2 infection. Am J Obstet Gynecol. ;232(1):123 e1– e12. Epub 20240403. (2025). 10.1016/j.ajog.2024.04.002. 38580043

[25] Moraes-Pinto M. I., Suano-Souza F. & Aranda C. S. Immune system: development and acquisition of immunological competence. J. Pediatr. (Rio J). 97 (Suppl 1), S59–S66. 10.1016/j.jped.2020.10.006 (2021). Epub 20201109. 33181111 PMC9432342

[26] Rechavi E. Timely and spatially regulated maturation of B and T cell repertoire during human fetal development. Sci. Transl Med. 7 (276), 276ra25. 10.1126/scitranslmed.aaa0072 (2015). 25717098

[27] Cardenas I. Viral infection of the placenta leads to fetal inflammation and sensitization to bacterial products predisposing to preterm labor. J. Immunol. 185 (2), 1248–1257. 10.4049/jimmunol.1000289 (2010). Epub 20100616. 20554966 PMC3041595

[28] Jennewein M. F., Abu-Raya B., Jiang Y., Alter G. & Marchant A. Transfer of maternal immunity and programming of the newborn immune system. Semin Immunopathol. ;39(6):605 – 13. Epub 20171002. (2017). 10.1007/s00281-017-0653-x. 28971246

[29] Cherayil B. J. & Jain N. From Womb to World: Exploring the Immunological Connections between Mother and Child. Immunohorizons 8 (8), 552–562. 10.4049/immunohorizons.2400032 (2024). 39172025 PMC11374749

[30] Gee S. The legacy of maternal SARS-CoV-2 infection on the immunology of the neonate. Nat. Immunol. 22 (12), 1490–1502. 10.1038/s41590-021-01049-2 (2021). Epub 20211006. 34616036

[31] Kim D. H. The association of maternal COVID-19-infection during pregnancy on the neonatal immune profile and associations with later diagnosis of neurodevelopmental disorders. Brain Behav. Immun. 123, 1071–1080 (2025). 39532198 10.1016/j.bbi.2024.11.014 PMC11684471

[32] CDC CfDCaP. About newborn screening 2024 [cited 2025]. Available from: https://www.cdc.gov/newborn-screening/about/index.html

[33] New York State Department of Health WC. Newborn Screening - Specimen Collection 2025 [cited 2025 September 24]. Available from: https://www.wadsworth.org/programs/newborn/screening/providers/specimen-collection

[34] Wik L. Proximity Extension Assay in Combination with Next-Generation Sequencing for High-throughput Proteome-wide Analysis. Mol. Cell. Proteom. 20, 100168. 10.1016/j.mcpro.2021.100168 (2021). Epub 20211027. PMC8633680 34715355

[35] Fredolini C. Proteome profiling of home-sampled dried blood spots reveals proteins of SARS-CoV-2 infections. Commun. Med. (Lond). 4 (1), 55. 10.1038/s43856-024-00480-4 (2024). Epub 20240402. 38565620 PMC10987641

[36] Dungu K. H. S. Proteomic profiling of neonatal herpes simplex virus infection on dried blood spots. Commun. Med. (Lond). 4 (1), 268. 10.1038/s43856-024-00711-8 (2024). Epub 20241218. 39695338 PMC11655519

[37] Broberg K. Evaluation of 92 cardiovascular proteins in dried blood spots collected under field-conditions: Off-the-shelf affinity-based multiplexed assays work well, allowing for simplified sample collection. Bioessays 43 (9), e2000299. 10.1002/bies.202000299 (2021). Epub 20210215. 33586222

[38] Proteomics, O. Data normalization and standardization. (2022).

[39] Proteomics, O. Multiplex analysis of inflammatory proteins: A comparative study across multiple platforms. (2022).

[40] WHO. Clinical management of COVID-19: interim guidance. Geneva: 2020 WHO/2019-nCoV/clinical/2020.5.

[41] Ritchie M. E. limma powers differential expression analyses for RNA-sequencing and microarray studies. Nucleic Acids Res. 43 (7), e47. 10.1093/nar/gkv007 (2015). Epub 20150120. 25605792 PMC4402510

[42] Rex D. A. B. A comprehensive pathway map of IL-18-mediated signalling. J. Cell. Commun. Signal. 14 (2), 257–266. 10.1007/s12079-019-00544-4 (2020). Epub 20191220. 31863285 PMC7272533

[43] Gerondakis S., Grossmann M., Nakamura Y., Pohl T. & Grumont R. Genetic approaches in mice to understand Rel/NF-kappaB and IkappaB function: transgenics and knockouts. Oncogene 18 (49), 6888–6895. 10.1038/sj.onc.1203236 (1999). 10602464

[44] Alvira C. M. Nuclear factor-kappa-B signaling in lung development and disease: one pathway, numerous functions. Birth Defects Res. Clin. Mol. Teratol. 100 (3), 202–216. 10.1002/bdra.23233 (2014). Epub 20140317. PMC4158903 24639404

[45] Perlin D. S. Levels of the TNF-Related Cytokine LIGHT Increase in Hospitalized COVID-19 Patients with Cytokine Release Syndrome and ARDS. mSphere. ;5(4). Epub 20200812. (2020). 10.1128/mSphere.00699-20. PMC7426176 32817460

[46] Sibilano R. A TNFRSF14-FcvarepsilonRI-mast cell pathway contributes to development of multiple features of asthma pathology in mice. Nat. Commun. 7, 13696. 10.1038/ncomms13696 (2016). Epub 20161216. PMC5171877 27982078

[47] Gupta A. SARS-CoV-2 infection- induced growth factors play differential roles in COVID-19 pathogenesis. Life Sci. 304, 120703. 10.1016/j.lfs.2022.120703 (2022). Epub 20220611. PMC9188443 35700841

[48] Gupta A., Qaisar R., Halwani R., Kannan M. & Ahmad F. TFPI and FXIII negatively and S100A8/A9 and Cystatin C positively correlate with D-dimer in COVID-19. Exp. Biol. Med. (Maywood). 247 (17), 1570–1576 (2022). Epub 20220620. doi: 10.1177/15353702221102117. 35723053 PMC9554165

[49] Perreau M. The cytokines HGF and CXCL13 predict the severity and the mortality in COVID-19 patients. Nat. Commun. 12 (1), 4888. 10.1038/s41467-021-25191-5 (2021). Epub 20210809. 34373466 PMC8352963

[50] Yanagita K. Hepatocyte growth factor may act as a pulmotrophic factor on lung regeneration after acute lung injury. J. Biol. Chem. 268 (28), 21212–21217 (1993). 8407957

[51] Xia C., Braunstein Z., Toomey A. C., Zhong J. & Rao X. S100 Proteins As an Important Regulator of Macrophage Inflammation. Front. Immunol. 8 10.3389/fimmu.2017.01908 (2017). Epub 20180105:1908. PMC5770888 29379499

[52] Hackney J. A. A myeloid program associated with COVID-19 severity is decreased by therapeutic blockade of IL-6 signaling. iScience 26 (10), 107813. 10.1016/j.isci.2023.107813 (2023). Epub 20230901. PMC10551843 37810211

[53] Lei H. A single transcript for the prognosis of disease severity in COVID-19 patients. Sci. Rep. 11 (1), 12174. 10.1038/s41598-021-91754-7 (2021). Epub 20210609. PMC8190311 34108608

[54] MacDonald L. COVID-19 and RA share an SPP1 myeloid pathway that drives PD-L1 + neutrophils and CD14 + monocytes. JCI Insight. ;6(13). Epub 20210618. (2021). 10.1172/jci.insight.147413. PMC8328085 34143756

[55] Mastboim N. S. An immune-protein score combining TRAIL, IP-10 and CRP for predicting severe COVID-19 disease. Cytokine 169, 156246. 10.1016/j.cyto.2023.156246 (2023). Epub 20230602. 37327532 PMC10235675

[56] Wagner K. I. Recruitment of highly cytotoxic CD8(+) T cell receptors in mild SARS-CoV-2 infection. Cell. Rep. 38 (2), 110214. 10.1016/j.celrep.2021.110214 (2022). Epub 20211217. PMC8677487 34968416

[57] Grau-Exposito J. Peripheral and lung resident memory T cell responses against SARS-CoV-2. Nat. Commun. 12 (1), 3010. 10.1038/s41467-021-23333-3 (2021). Epub 20210521. 34021148 PMC8140108

[58] Laing A. G. A dynamic COVID-19 immune signature includes associations with poor prognosis. Nat. Med. 26 (10), 1623–1635. 10.1038/s41591-020-1038-6 (2020). Epub 20200817. 32807934

[59] Gruber C. N. Mapping Systemic Inflammation and Antibody Responses in Multisystem Inflammatory Syndrome in Children (MIS-C). Cell. ;183(4):982 – 95 e14. Epub 20200914. (2020). 10.1016/j.cell.2020.09.034. 32991843 PMC7489877

[60] Mahalingam S. Chemokines and viruses: friends or foes? Trends Microbiol. 11 (8), 383–391. 10.1016/s0966-842x(03)00157-4 (2003). 12915096

[61] Blot M. The dysregulated innate immune response in severe COVID-19 pneumonia that could drive poorer outcome. J. Transl Med. 18 (1), 457. 10.1186/s12967-020-02646-9 (2020). Epub 20201203. 33272291 PMC7711269

[62] Li M. Elevated Exhaustion Levels of NK and CD8(+) T Cells as Indicators for Progression and Prognosis of COVID-19 Disease. Front. Immunol. 11, 580237. 10.3389/fimmu.2020.580237 (2020). Epub 20201014. PMC7591707 33154753

[63] Paranga T. G. Distinct soluble immune checkpoint profiles characterize COVID-19 severity, mortality and SARS-CoV-2 variant infections. Front. Immunol. 15, 1464480 (2024). 10.3389/fimmu.2024.1464480 PMC11456479 39376569

[64] Blanco J. R. Elevated levels of serum CDCP1 in individuals recovering from severe COVID-19 disease. Aging (Albany NY). 14 (4), 1597–1610 (2022). Epub 20220216. doi: 10.18632/aging.203898. 35172279 PMC8908919

[65] Ruiz A. OPG and BAFF as predictive biomarkers of the severity of SARS-CoV-2 infection. J. Cell. Mol. Med. 29 (3), e70189. 10.1111/jcmm.70189 (2025). PMC11783147 39888266

[66] Wu X. Damage to endothelial barriers and its contribution to long COVID. Angiogenesis 27 (1), 5–22. 10.1007/s10456-023-09878-5 (2024). Epub 20230427. 37103631 PMC10134732

[67] Salomao R. Involvement of Matrix Metalloproteinases in COVID-19: Molecular Targets, Mechanisms, and Insights for Therapeutic Interventions. Biology (Basel). ;12(6). Epub 20230610. (2023). 10.3390/biology12060843. PMC10295079 37372128

